# Alcohol Intake and Prevalent Kidney Stone: The National Health and Nutrition Examination Survey 2007–2018

**DOI:** 10.3390/nu16172928

**Published:** 2024-09-01

**Authors:** Sandipan Shringi, Christina A. Raker, Michel Chonchol, Jie Tang

**Affiliations:** 1 Division of Kidney Diseases and Hypertension, Alpert Medical School of Brown University, Providence, RI 02903, USA; sandipan.shringi@brownphysicians.org; 2 Lifespan Biostatistics, Epidemiology, Research Design, and Informatics Core, Providence, RI 02903, USA; craker@lifespan.org; 3 Division of Kidney Diseases and Hypertension, University of Colorado School of Medicine, Aurora, CO 80045, USA; michel.chonchol@cuanschutz.edu; 4 Division of Kidney Diseases and Hypertension, Department of Medicine, Brown Physicians Inc., 375 Wampanoag Trail, East Providence, RI 02915, USA

**Keywords:** alcohol intake, beer, wine, liquor, kidney stone

## Abstract

The association of alcohol intake with kidney stone disease (KSD) is not clear based on current clinical evidence. We examined the National Health and Nutrition Examination Survey (NHANES) 2007–2018 and used logistic regression analyses to determine the independent association between alcohol intake and prevalent KSD. In total, 29,684 participants were eligible for the final analysis, including 2840 prevalent stone formers (SFs). The mean alcohol intake was 37.0 ± 2.4 g/day among SFs compared to 42.7 ± 0.9 among non-SFs (*p* = 0.04). Beer [odds ratio (OR) = 0.76, 95% CI: 0.61–0.94, *p* = 0.01] and wine (OR = 0.75, 95% CI: 0.59–0.96, *p* = 0.03) intakes were strongly associated with lower odds of prevalent KSD, while liquor intake had no association. Furthermore, the effects of beer and wine intakes on stone formation were dose-dependent. The OR for comparing participants drinking 1–14 g/day of beer to non-drinkers was 1.41 (95%CI: 0.97–2.05, *p* = 0.07), that of >14–≤28 g/day of beer to non-drinkers was 0.65 (95% CI: 0.42–1.00, *p* = 0.05), that of >28–≤56 g/day of beer to non-drinkers was 0.60 (95% CI: 0.39–0.93, *p* = 0.02), and that of >56 g/day of beer to non-drinkers was 0.34 (95% CI: 0.20–0.57, *p* < 0.001). Interestingly, the effect of wine intake was only significant among participants drinking moderate amounts (>14–28 g/day), with an OR of 0.54 (95% CI: 0.36–0.81, *p* = 0.003) compared to non-drinkers, but this effect was lost when comparing low-level (1–14 g/day) and heavy (>28 g/day) wine drinkers to non-drinkers. These effects were consistent in spline models. This study suggests that both moderate to heavy beer intake and moderate wine intake are associated with a reduced risk of KSD. Future prospective studies are needed to clarify the causal relationship.

## 1. Introduction

Kidney stone disease (KSD) is highly prevalent and affects 10% of the US population [1]. It is a systemic illness affecting major organs like the heart, kidney, and bone, while being strongly associated with diabetes, hypertension, and dyslipidemia, and carries a significant economic burden [2]. Among different types of kidney stones (KSs), calcium-based stones account for the vast majority [3]. The process of stone formation starts with an increasing concentration of stone-forming elements, which eventually surpass supersaturation, followed by nucleation and growth. The whole process is affected by an intricate interplay between stone promoters and inhibitors. As diet directly affects several key KS promoters and inhibitors, e.g., calcium, oxalate, uric acid, magnesium, and citric acid, dietary modification is considered the most effective preventive measure for KSD, with adequate hydration being a key component directly affecting supersaturation. Different types of liquid may also have different modifying effects on KS risk: lemon juice, tea, and coffee intake have been associated with a lower risk while apple juice and soda intake have been associated with a higher risk [4,5,6,7,8].

On one hand, alcohol (a unique type of liquid) may increase urinary concentrations of calcium [9,10,11], phosphorus [11], and uric acid [11,12,13,14,15], thereby increasing their supersaturations. On the other hand, it may also increase the urinary concentration of magnesium [11,16,17,18,19], which lowers the saturation [20], nucleation, and growth rates of calcium oxalate crystals [21] and inhibits the adhesion of these crystals to kidney cells [22]. Furthermore, it contains a fair amount of liquid and has a diuretic effect [23,24,25,26], which can reduce KS formation. Therefore, the effects of alcohol intake on KSD are conflicting, with this complication being noted in previous studies. Hirvonen et al. [27] reported a reduced KS risk from alcohol consumption in a large Finnish male cohort. However, Zhou et al. [28], in their examination of the NHANES 2007–2016 cohort, did not find any significant associations between alcohol intake and KSD, even after adjusting for confounders. Regarding the specific types of alcohol, Krieger et al. [29] found that men drinking beer had a 53% reduced risk of stone formation compared to their matched controls. Interestingly, Goldfarb et al. [30] reported a unique dose effect from beer intake. They found that moderate beer drinking was associated with a reduced risk of KSD, while low and heavy drinking had no associations. Studies by Curhan et al. [5] and Ferraro et al. [8] both showed a reduced risk of KS from drinking wine, but Goldfarb et al. [30] failed to find such an association. Drinking liquor did not appear to affect KS risk according to three previous clinical studies [5,8,30], but Wang et al. [31] reported a significant reduction in KS risk from liquor exposure in a large Chinese population. All these studies were limited by the inadequate adjustment of potential confounders for KSD.

Due to the remaining uncertainty regarding the effect of alcohol intake on KS risk, we examined a large US population survey database, the National Health and Nutrition Examination Survey (NHANES) from 2007 to 2018, to examine the independent association between alcohol intake and KSD.

## 2. Methods

### 2.1. Study Population

**Figure 1:**
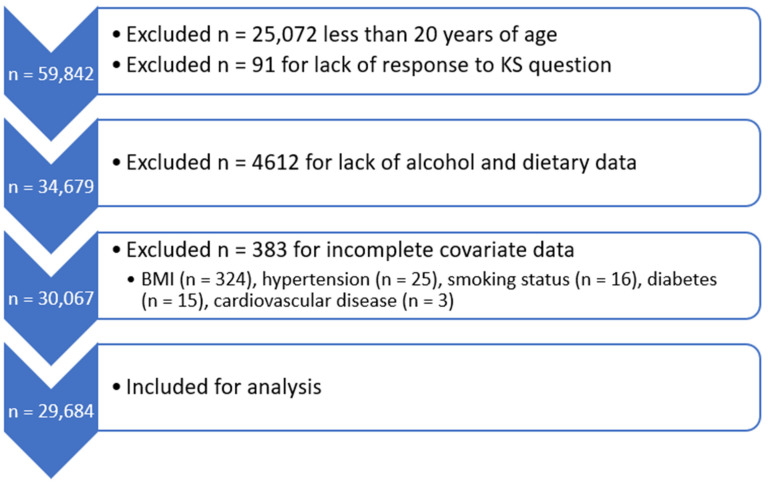
Selection of study population.

NHANES is an ongoing program of nationally representative cross-sectional health surveys and physical examinations of adults and children in the US. The program has been conducted continuously in two-year cycles since 1999. The survey component collects demographic, socioeconomic, dietary, and health-related information. The examination component, conducted by trained medical personnel, obtains physiological measurements and data from laboratory testing. A total of 59,842 participants were interviewed for NHANES from 2007 to 2018. Of these, our analysis included 29,684 participants aged 20 years or older with complete data on alcohol intake, history of KS, and the covariates of interest (Figure 1).

### 2.2. Primary Exposure and Outcome

Our primary exposure was the amount and type of alcohol intake using individual foods and beverages reported during the 24 h dietary recall interviews. Briefly, each food reported by a participant was assigned a code for linkage to ingredients and nutrient composition databases maintained by the US Department of Agriculture’s Food Surveys Research Group. This information, along with the amount consumed, is stored in the Individual Foods file for each of the two recall interviews (DR1IFF, DR2IFF) and is the basis for estimating dietary intake in the Total Nutrient Intake files (DR1TOT, DR2TOT). Foods with a non-zero amount of alcohol were selected from the Individual Foods files and linked to the Food Code Descriptions files (DRXFCD) to classify each entry as beer, wine, liquor, mixed drinks, and other sources of alcohol, such as prepared food containing alcohol. The total alcohol in grams by type was summed for each participant. Alcohol drinking status was obtained by questionnaire and categorized as never, former (no drinks in the past 12 months), and current (at least one drink in the past 12 months). Never and former drinkers who reported any alcohol in the 24 h recall interviews were excluded. Only data from day one out of the two 24 h recall periods was included in the present analysis.

### 2.3. Primary Outcome

The outcome or dependent variable of interest was prevalent KS disease. This was extracted from the interview data file. ‘Have you ever had a kidney stone?’ was the question asked during the standardized home interview. Adult participants who responded ‘yes’ to the question were considered to have a history of KS.

### 2.4. Covariates

Age, sex, race, history of diabetes, hypertension, thiazide use, and smoking status was obtained from the questionnaire. Body mass index (BMI) was calculated from height and weight measured during the health examination. Information on total intakes of calories, protein, and fluids (excluding alcohol), along with dietary intakes of sodium, potassium, and calcium, were obtained from the total nutrient intake data file from the same day-one interview when data on the type and amount of alcohol intake were collected.

### 2.5. Analysis

Statistical analysis was performed with Stata MP 18 (Stata Corp, College Station, TX, USA). The complex sampling design was incorporated by applying strata, primary sampling units, and sampling weights via survey-specific procedures. Day one 24 h recall weights were used for all analyses. Logistic regression was used to estimate unadjusted and multivariable-adjusted odds ratios (ORs) and 95% confidence intervals (CIs) for alcohol intake and prevalent KSD. Alcohol intake was examined as both a categorical and a continuous predictor of KS formation. Categories were created from the type of alcohol consumed and, within each type, from amounts reflecting the ratios of a standard drink (14 g) [32]. Alcohol intake of each type was also examined by including restricted cubic splines in the regression model [33]. Knots were specified at the 5th, 25th, 50th, 75th, and 95th percentiles of the distribution among participants drinking at least 1 g of a specific type of alcohol. A binary indicator variable was added to the model to represent 0 to less than 1 g of alcohol [34]. Deviations from linearity were assessed by testing coefficients for non-linear spline terms. The multivariable models included age (years), sex, race (non-Hispanic White, non-Hispanic Black, Hispanic/Latino, non-Hispanic other), BMI (<25, 25–<30, >30 kg/m^2^), diabetes (no, borderline/yes), hypertension, thiazide diuretic use, smoking (never, former, current), total dietary calories (kcal), total dietary protein (g), total fluid without alcohol contribution (g), dietary sodium (mg), potassium (mg), and calcium (mg). All *p*-values presented were two-tailed, with *p* < 0.05 considered statistically significant.

## 3. Results

A total of 29,684 participants were included in this analysis. In total, 2840 (9.7%) of these reported a history of stones. Among stone formers, 2087 (78.1%) participants reported drinking alcohol currently, as compared to 19,985 (79.6%) participants among non-stone formers (*p* = 0.002). Mean alcohol intake was also significantly lower, with 37.0 ± 2.4 g/day in stone formers compared to 42.7 ± 0.91 g/day in non-stone formers (*p* = 0.04). Stone formers tended to be older, predominantly male, and non-Hispanic White, with a higher BMI compared to non-stone formers. They were also more likely to have a history of diabetes and hypertension, to use thiazides, and to have a history of smoking (Table 1).

Among current drinkers, 235 (43.8%) stone formers drank beer, 103 (22.5%) drank wine, and 110 (24.8%) drank liquor, as compared to 2828 (43.5%) non-stone formers who drank beer, 1209 (23.3%) who drank wine, and 1103 (17.9%) who drank liquor.

In a univariate analysis of alcohol types, the consumption of only beer or only wine was associated with lower odds of prevalent KS when compared to never drinkers or current drinkers who did not report alcohol by dietary recall. These associations remained after adjustment for age, sex, race, BMI, histories of hypertension, diabetes, thiazide use, cigarette smoking, dietary intakes of calories, protein, fluid without alcohol contribution, sodium, potassium, and calcium (Table 2).

We also evaluated KS risk among current drinkers with exclusive intakes of alcohol modeled as a categorical variable in tertiles or quartiles after rounding to the nearest multiple of one standard drink (14 g). The multivariate-adjusted OR for stone formation among participants drinking 1–≤14 g/day of beer was 1.41 (95% CI: 0.97–2.05), while that for >14–28 g/day was 0.65 (95% CI: 0.42–1.00), >28–56 g/day was 0.60 (95% CI: 0.39–0.93), and >56 g/day was 0.34 (95% CI: 0.20–0.57) compared to those who did not drink beer (Table 3).

Interestingly, the multivariate-adjusted OR for stone formation was 1.14 (95% CI: 0.72–1.83) among participants drinking 1–≤14 g/day of wine, 0.54 (95% CI: 0.36–0.81) among those drinking >14–28 g/day, and 0.85 (95% CI: 0.54–1.33) among those drinking >28 g/day compared to those who did not drink wine (Table 4), showing a unique effect of moderate wine intake on KS risk.

**Figure 2:**
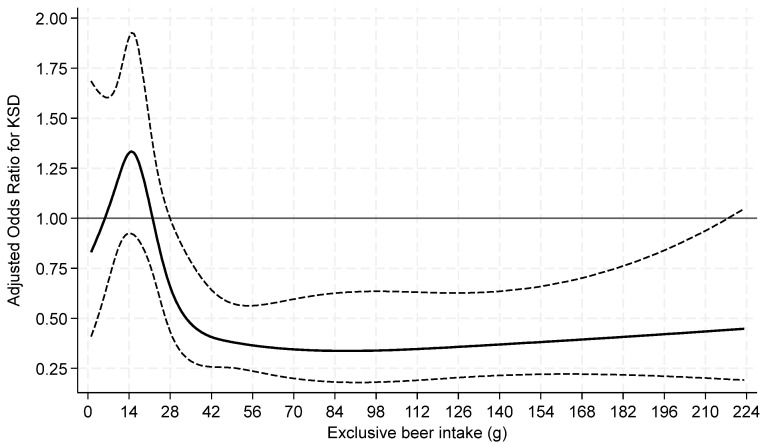
Odds ratios of prevalent kidney stone by restricted cubic splines for exclusive **beer** intake **among current drinkers**. Knots at 9.4, 14.9, 28.1, 47.95, and 126.4 g of beer with a binary indicator variable for 0–<1 g. *p* = 0.02 for test of linearity. The x-axis was truncated at 224 g, omitting 27 respondents with intakes >224–832 g.

**Figure 3:**
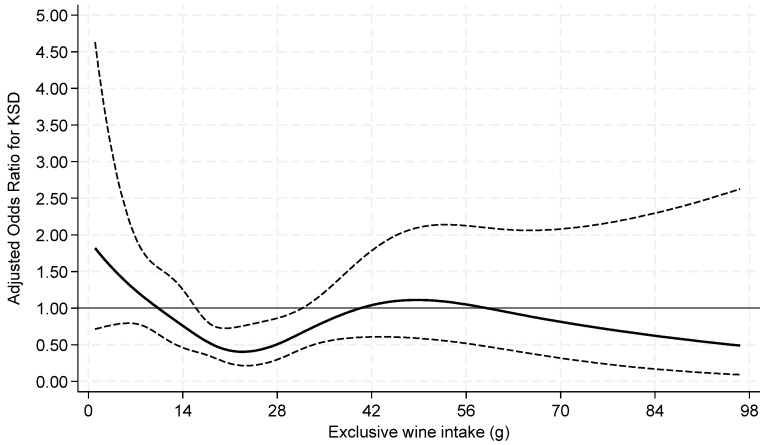
Odds ratios of prevalent kidney stone by restricted cubic splines for exclusive **wine** intake **among current drinkers**. Knots at 7.2, 15.1, 21.6, 31.2, and 63.6 g of wine with a binary indicator variable for 0–<1 g. *p* = 0.04 for test of linearity. The x-axis was truncated at 98 g, omitting 14 respondents with intakes >98–167 g.

When beer and wine consumption were examined as continuous variables by restricted cubic splines, both exhibited non-linear associations with prevalent KS (Figure 2 and Figure 3).

**Figure 4:**
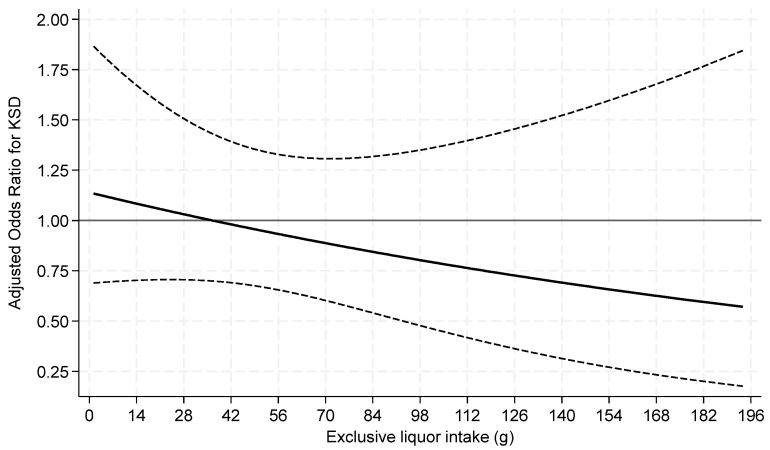
Odds ratios of prevalent kidney stone for continuous **liquor** intake **among current drinkers**. *p* = 0.29 for test of linearity. The x-axis was truncated at 196 g, omitting 17 respondents with intakes >196–404 g.

Exclusive liquor consumption was not associated with prevalent KS when analyzed as a categorical or continuous variable (Table 5 and Figure 4).

## 4. Discussion

Alcohol intake has been implicated in many health problems, including cardiovascular disease, liver damage, cancer, and behavioral disorders. Its role in KS formation, however, remains unclear. Here, we analyzed a large cohort of a US population and showed a strong protective effect from beer and wine intakes on the odds of prevalent KS. To the best of our knowledge, this is the largest population study examining specifically the role and type of alcohol intake on the risk of KS formation independent of other known confounders.

KS formation occurs when stone forming elements, most commonly calcium, phosphorus, oxalate, and uric acid reach a supersaturation point followed by nucleation, aggregation, and growth. While some, including citric acid and magnesium [22,35,36], inhibit KS formation, others including zinc [37,38,39,40,41] have been implicated as promoters. Alcohol intake, in turn, has been shown to either directly affect the concentrations of stone forming elements or to indirectly affect their promoters and inhibitors, thereby potentially playing a role in KS formation. Alcohol intake can lead to suppressed osteoblast activity [42] and increased osteoclastic activity [43], thereby leading to bone loss and increasing urinary calcium [9,10,11] and phosphorus [11]. Together, these effects seem to increase the risk of KS formation. However, it also promotes urinary magnesium excretion [11,16,17,18,19,44] and increases urine output [23,24,25,26]; both can be protective against stone formation. The effect of these biochemical changes on urine constituents has raised uncertainty regarding the overall effect of alcohol intake on KS risk.

Beer has a limited alcohol content, but contains a large amount of guanosine [45], which is metabolized to uric acid [46,47]. As a result, a higher beer intake may lead to an increased urinary excretion of uric acid and may promote KS formation [15,45,46]. While the effect of purine in beer on stone risk has not been studied exclusively, water, which accounts for 95% of the content of beer [32], is known to reduce the supersaturations of stone forming elements and crystal formation [48,49]. Indeed, optimal water intake is proven to be an effective intervention for KS prevention [50,51]. We found that beer intake is strongly associated with a reduced risk of prevalent KS by as much as 24%. Our findings are consistent with what has been previously reported from other large population-based studies [5,8,27]. It should be noted that our study found a direct dose–response relationship between the number of standard drinks consumed and KS risk, with participants drinking moderate (>14–28 g) to large (>28–56 g) amounts of beer every day having a progressively lowered risk of KS formation. This protective effect is likely not driven simply by the high water content in beer, as the strong association between beer intake and prevalent KS persisted after we adjusted for water content from beer in the sensitivity analysis (Supplementary Table S1). Indeed, beer contains a wide range of ingredients, such as hops, which can prevent calcium loss from bone [52,53], inhibit calcium oxalate crystal formation, and dissolve kidney stones [54]. It may also contain barley, which has been used for the prevention of KS, as it is not only rich in magnesium but also has diuretic and urine alkalinization effects that modify KS risk [55]. Further studies are needed to examine the independent effects of these specific components on KS risk.

Despite having high water content, wine has unique features that may affect KS risk. Wine consumers tend to have a higher excretion of urinary calcium [56], phosphorus [56], zinc [57], and a lower urinary excretion of magnesium [56] compared to liquor consumers, and this effect remained in people consuming dealcoholized wine [56], suggesting a role of congeners in wine affecting KS risk. In fact, each glass of wine can not only provide protective minerals such as magnesium and potassium, but it also contains calcium, phosphorus, and zinc, which can potentially increase KS risk. Wine also contains phenolic compounds which can dissolve calcium-based stones and inhibit the growth of KSs [58,59,60]. Even though the alcohol component has an aquaresis effect [23,24,25,26], the volume of wine consumed is usually low. Therefore, the effect of wine consumption on KS risk is unclear. In this study, we found a 25% reduction in KS risk among participants who drank wine. But this protection appeared to be modified by the amount of wine intake. Drinking moderate amounts (14 g–28 g/day) of wine was associated with reduced risk of KSD, whereas no protective effects were found among low (<14 g/day) or heavy (>28 g/day) drinkers. suggesting a U-shaped response. Previous studies have reported a reduced risk of prevalent KS formation by 39% in men [5] and 59% in women [61], and this was confirmed by Ferraro et al. [8] in a prospective study. However, Curhan et al. also reported a dose-related linear reduction in risk of KS in both male [5] and female [61] wine drinkers. The cause of this discrepant finding among higher wine drinkers is not clear. It might reflect the differences in study cohort and analysis methods. In addition, the studies by Curhan et al. did not adjust for dietary fluid intake. Lastly, the regression analyses in this study included many other essential confounders of KSD. Since the water content in wine can be as high as 88% [32], and there are many other unique ingredients in wine [62], it is feasible that moderate wine drinking is associated with a more favorable balance between stone promoting factors and inhibitors.

Liquor, which is a concentrated form of ethanol, leads to increased urinary calcium [9,10,11], phosphorus [11], and uric acid [63], raising the possibility of harmful effect on KS formation. However, it also leads to increased urinary magnesium [11,16,17,18,19,44] and while low in volume itself when consumed, it does lead to the suppression of vasopressin [64,65], which in turn results in increases urine volume, suggesting a protective role in KS formation. We found that drinking any amount of liquor has no association with risk of prevalent KSD, reflecting a well-balanced effect from KS promoters and inhibitors. Our finding is consistent with what has been reported by Goldfarb et al. in a study of the Vietnam-Era Twin Registry [30]. However, Wang et al. [31], in their large population study, found that drinking any amount of liquor had a reduced risk of prevalent KS. This discrepancy could be due to the differences in study cohorts.

Our study has limitations. First, this is a cross-sectional study, and conclusions regarding causal or temporal relationships cannot be made. Second, it is possible that stone formers who are aware of their disease increase their alcohol intake to increase fluid volume. However, such practice is generally not recommended due to the lack of solid clinical evidence and concerns of the overall negative impact to health, especially considering a high prevalence of hypertension among KS formers. Third, KS diagnosis was self-reported during interviews. Therefore, misclassification is possible, as stone formers may have a recall bias and some may not be aware that they had a stone. Furthermore, alcohol consumers may underestimate and under-report the amount of alcohol consumed. Regardless, this should be biased toward null. Finally, we also could not evaluate the effect of alcohol intake on urinary risk profile or the type of stone, since these data were not available in the NHANES.

## 5. Conclusions

Our study demonstrated that moderate to high beer intake and moderate wine intake are associated with a reduced prevalence of KS disease. Future prospective studies are needed to clarify the causal relationship and underlying mechanisms.

## Figures and Tables

**Table 1:** Baseline characteristics of study population.

	KS Former	Non-KS Former	*p* Value
Total n, unweighted	9.7 (2840)	90.3 (26,844)	
Male sex	54.6 (1571)	47.4 (12,865)	<0.001
Age (y)	53.7 ± 0.38	46.8 ± 0.26	<0.001
Race			<0.001
Non-Hispanic White	76.2 (1553)	65.3 (10,851)	
Non-Hispanic Black	5.9 (376)	11.8 (5995)	
Hispanic/Latino	11.7 (690)	14.7 (6844)	
Non-Hispanic other	6.1 (221)	8.2 (3154)	
BMI (kg/m^2^)			<0.001
<25.0	19.8 (542)	30.2 (7803)	
25.0–<30.0	32.9 (962)	32.8 (8784)	
30.0+	47.2 (1336)	37.0 (10,257)	
History of diabetes	22.4 (736)	10.8 (3895)	<0.001
History of hypertension	48.7 (1487)	32.3 (9841)	<0.001
Thiazide diuretic use	12.6 (377)	7.8 (2498)	<0.001
Smoking status			<0.001
Never	49.8 (1392)	56.2 (15,104)	
Former	30.6 (884)	24.0 (6267)	
Current	19.6 (564)	19.8 (5473)	
Total calories (kcal)	2122.9 ± 28.7	2142.9 ± 8.9	0.5
Protein intake (g)	80.8 ± 1.4	82.8 ± 0.42	0.16
Dietary sodium (mg)	3534.0 ± 54.6	3539 ± 15.9	0.93
Dietary potassium (mg)	2644.0 ± 38.3	2690.2 ± 15.5	0.22
Dietary calcium (mg)	934.3 ± 15.3	973.7 ± 6.6	0.02
Total fluid intake, excluding alcohol (g)	2905.0 ± 35.4	2885.9 ± 20.0	0.58
Alcohol drinking status			0.002
Never	12.3 (442)	13.6 (4638)	
Former (0 drinks in past year)	9.7 (311)	6.9 (2221)	
Current (>0 drinks in past year)	78.1 (2087)	79.6 (19,985)	
Type of alcohol, if any			0.01
Beer only	43.8 (235)	43.5 (2828)	
Wine only	22.5 (103)	23.3 (1209)	
Liquor only	24.8 (110)	17.9 (1103)	
Other/combination	8.9 (69)	15.3 (946)	

Values are expressed as weighted means ± SE or % (unweighted n). Abbreviations: BMI = body mass index, KS = kidney stone.

**Table 2:** Odds ratios of prevalent kidney stone according to type of alcohol.

	Unadjusted Model		Adjusted Model 1		Adjusted Model 2		Adjusted Model 3	
	OR (95% CI)	*p* Value	OR (95% CI)	*p* Value	OR (95% CI)	*p* Value	OR (95% CI)	*p* Value
Never/Currently none	REF		REF		REF		REF	
Beer only	0.79 (0.64–0.97)	0.02	0.76 (0.62–0.94)	0.01	0.79 (0.64–0.97)	0.03	0.76 (0.61–0.94)	0.01
Wine only	0.75 (0.58–0.99)	0.04	0.64 (0.49–0.84)	0.001	0.74 (0.57–0.96)	0.02	0.75 (0.59–0.96)	0.03
Liquor only	1.08 (0.77–1.52)	0.63	1.04 (0.74–1.47)	0.82	1.05 (0.75–1.49)	0.76	0.99 (0.69–1.42)	0.97

Abbreviations: OR = odds ratio, CI = confidence interval. REF = never drinkers and current drinkers who did not report alcohol intake on day 1 recall. Model 1: adjusted for demographics. Model 2: adjusted for BMI, histories of hypertension, diabetes, thiazide use, cigarette smoking, in addition to model 1. Model 3: adjusted for dietary intakes of calories, protein, fluid (minus alcohol contribution), sodium, potassium, and calcium, in addition to model 2.

**Table 3:** Odds ratios of prevalent kidney stone according to exclusive **beer** intake **among current drinkers**.

	Unadjusted Model		Adjusted Model 1		Adjusted Model 2		Adjusted Model 3	
Beer	OR (95% CI)	*p* Value	OR (95% CI)	*p* Value	OR (95% CI)	*p* Value	OR (95% CI)	*p* Value
0–<1 g	REF		REF		REF		REF	
1–≤14 g	1.46 (1.00–2.15)	0.05	1.35 (0.93–1.96)	0.11	1.45 (1.00–2.12)	0.05	1.41 (0.97–2.05)	0.07
>14–28 g	0.67 (0.44–1.01)	0.06	0.65 (0.43–0.99)	0.04	0.66 (0.43–1.02)	0.06	0.65 (0.42–1.00)	0.05
>28–56 g	0.60 (0.40–0.91)	0.02	0.61 (0.40–0.92)	0.02	0.64 (0.41–0.98)	0.04	0.60 (0.39–0.93)	0.02
>56 g	0.39 (0.24–0.63)	<0.001	0.38 (0.24–0.62)	<0.001	0.38 (0.24–0.62)	<0.001	0.34 (0.20–0.57)	<0.001

Abbreviations: OR = odds ratio, CI = confidence interval. REF = No alcohol intake on day 1 recall or less than 1 g of beer only. Model 1: adjusted for demographics. Model 2: adjusted for BMI, histories of hypertension, diabetes, thiazide use, and cigarette smoking, in addition to model 1. Model 3: adjusted for dietary intakes of calories, protein, fluid (minus alcohol contribution), sodium, potassium, and calcium, in addition to model 2. In total, 18,532 records were included.

**Table 4:** Odds ratios of prevalent kidney stone according to exclusive **wine** intake **among current drinkers**.

	Unadjusted Model		Adjusted Model 1		Adjusted Model 2		Adjusted Model 3	
Wine	OR (95% CI)	*p* Value	OR (95% CI)	*p* Value	OR (95% CI)	*p* Value	OR (95% CI)	*p* Value
0–<1 g	REF		REF		REF		REF	
1–≤14 g	0.98 (0.62–1.53)	0.91	0.90 (0.56–1.44)	0.66	1.10 (0.69–1.77)	0.68	1.14 (0.72–1.83)	0.57
>14–28 g	0.52 (0.35–0.77)	0.001	0.43 (0.29–0.64)	<0.001	0.53 (0.36–0.78)	0.002	0.54 (0.36–0.81)	0.003
>28 g	0.86 (0.52–1.44)	0.56	0.77 (0.47–1.27)	0.31	0.87 (0.54–1.4)	0.56	0.85 (0.54–1.33)	0.47

Abbreviations: OR = odds ratio, CI = confidence interval. REF = No alcohol intake on day 1 recall or less than 1 g of wine only. Model 1: adjusted for demographics. Model 2: adjusted for BMI, histories of hypertension, diabetes, thiazide use, and cigarette smoking, in addition to model 1. Model 3: adjusted for dietary intakes of calories, protein, fluid (minus alcohol contribution), sodium, potassium, and calcium, in addition to model 2. In total, 16,781 records were included.

**Table 5:** Odds ratios of prevalent kidney stone according to exclusive **liquor** intake **among current drinkers**.

	Unadjusted Model		Adjusted Model 1		Adjusted Model 2		Adjusted Model 3	
Liquor	OR (95% CI)	*p* Value	OR (95% CI)	*p* Value	OR (95% CI)	*p* Value	OR (95% CI)	*p* Value
0–<1 g	REF		REF		REF		REF	
1–≤28 g	1.18 (0.70–1.99)	0.53	1.15 (0.67–1.96)	0.62	1.20 (0.71–2.02)	0.49	1.16 (0.69–1.97)	0.58
>28 g	0.97 (0.65–1.44)	0.87	0.94 (0.62–1.40)	0.75	0.94 (0.62–1.43)	0.79	0.85 (0.56–1.30)	0.45

Abbreviations: OR = odds ratio, CI = confidence interval. REF = No alcohol intake on day 1 recall or less than 1 g of liquor only. Model 1: adjusted for demographics. Model 2: adjusted for BMI, histories of hypertension, diabetes, thiazide use, and cigarette smoking, in addition to model 1. Model 3: adjusted for dietary intakes of calories, protein, fluid (minus alcohol contribution), sodium, potassium, and calcium, in addition to model 2. In total, 16,682 records were included.

## Data Availability

Records and data pertaining to this study are stored electronically at the Division of Kidney Diseases and Hypertension, Alpert Medical School of Brown University in Providence, USA, and can be provided by the corresponding author on a reasonable request.

## Supplementary Materials

The following supporting information can be downloaded at: https://www.mdpi.com/article/10.3390/nu16172928/s1, Table S1: Multivariable regression models adjusting for total fluid intake (with contribution from alcohol added).
