# Supplementary material for: Alcohol Intake and Prevalent Kidney Stone: The National Health and Nutrition Examination Survey 2007–2018
Source: Nutrients. 2024 Sep 1;16(17):2928. doi: 10.3390/nu16172928 (PMC11397207; doi:10.3390/nu16172928)
Supplement: Supplementary file 1 [file nutrients-16-02928-s001.zip › nutrients-3142935-supplementary.pdf]

7/17/2024

Supplementary Table S1. Multivariable regression models adjusting for total fluid intake (with contribution from alcohol added).

	Adjusted OR (95% CI)	p value
Model 1: Type of alcohol		
Never/Currently none	REF	
Beer only	0.76 (0.60-0.95)	0.02
Wine only	0.75 (0.58-0.96)	0.02
Liquor only	0.99 (0.69-1.42)	0.97
Model 2: Beer intake only		
0-<1g	REF	
1-≤ 14g	1.41 (0.97-2.04)	0.07
>14-28g	0.64 (0.42-0.99)	0.045
>28-56g	0.59 (0.39-0.91)	0.02
>56g	0.32 (0.19-0.55)	<0.001
Model 3: Wine intake only		
0-<1g	REF	
1-≤ 14g	1.15 (0.72-1.83)	0.57
>14-28g	0.54 (0.36-0.81)	0.003
>28g	0.84 (0.53-1.33)	0.46
Model 4: Liquor intake only		
0-<1g	REF	
1-≤ 28g	1.16 (0.68-1.96)	0.58
>28g	0.85 (0.56-1.29)	0.44

Models were adjusted for demographics, BMI, histories of hypertension, diabetes, thiazide use, cigarette smoking, and dietary intakes of calories, protein, fluid from all sources, sodium, potassium, and calcium.
